# Metabolomics characterizes the metabolic changes of Lonicerae Japonicae Flos under different salt stresses

**DOI:** 10.1371/journal.pone.0243111

**Published:** 2020-12-01

**Authors:** Zhichen Cai, Huan Chen, Jiajia Chen, Rong Yang, Lisi Zou, Chengcheng Wang, Jiali Chen, Mengxia Tan, Yuqi Mei, Lifang Wei, Shengxin Yin, Xunhong Liu

**Affiliations:** College of Pharmacy, Nanjing University of Chinese Medicine, Nanjing, China; Hainan University, CHINA

## Abstract

Salt stress affects the metabolic homeostasis of medicinal plants. However, medicinal plants are sessile organisms that cannot escape from salt stress. They acclimatize themselves to the stress by reprogramming their metabolic pathways. Lonicerae Japonicae Flos (LJF) with strong antioxidant activity is commonly used in traditional Chinese medicine, tea, and beverage. Nevertheless, the variation of integrated metabolites in LJF under different salt stresses remains unclear. In this study, High Performance Liquid Chromatography tandem triple time-of-flight mass spectrometry (HPLC- triple TOF-MS/MS) coupled with multivariate statistical analysis was applied to comparatively investigate the metabolites changes in LJF under different salt stress (0, 100, 200, 300 mM NaCl). Total 47 differential metabolites were screened from 79 metabolites identified in LJF under different salt stress. Low salt-treated group (100 mM NaCl) appeared to be the best group in terms of relative contents (peak areas) of the wide variety in bioactive components. Additionally, the phenylpropanoid pathway, monoterpenoid biosynthesis, glycolysis, TCA cycle, and alkaloid biosynthesis were disturbed in all salt-stress LJF. The results showed that LJF metabolisms were dramatically induced under salt stress and the quality of LJF was better under low salt stress. The study provides novel insights into the quality assessment of LJF under salt stress and a beneficial framework of knowledge applied to improvement the medicinal value of LJF.

## Introduction

The growth, development, and production of medicinal plant are usually affected by various environmental conditions including salinity [1, 2], drought [3], nutrient [4], cold [5], abiotic stress and biotic stress [6]. Soil salinity is the most common stress that can stimulate the accumulation of compatible osmolytes and antioxidants [7]. Primary metabolites including fatty acids, amino acids, and nucleotides are synthesized by all plant species and play important roles in the life cycle of plants [8]. As an autotrophic organism, plant can accumulate and synthesize specialized metabolites, such as phenolic, flavonoid, terpenoid, and other secondary metabolites to acquire a competitive advantage under environmental stresses. However, the accumulated and synthesized of secondary metabolites in medicinal plants under salt stress are still poorly known. Phenolic compounds are secondary metabolites that possess the high antioxidant capacity to protect plants from oxidative damage under abiotic stress [9]. Up to now, the functions of phenolic compounds on the adaptation of plants to the environment are still elusive. Understanding the alterations of plant metabolome under salt stress could provide potential clues to improve salt tolerance and quality in plants.

The perennial twining woody vine *Lonicera japonica* Thunb. (Caprifoliaceae) is cultivated worldwide as an ornamental plant and commonly used in traditional Chinese and Japanese medicine. Its flower buds are listed as the top grade in ‘Ming Yi Bie Lu’ and recorded in Chinese pharmacopeia as Lonicerae Japonicae Flos (LJF). LJF has been used thousands of years for the treatment of fever, infections, sores, and swelling. Modern pharmacological studies also prove that it possesses antiviral, anti-inflammatory, antioxidant, antitumor, and hepatoprotective activities [10–12]; and bioactive compounds including phenolic acids, flavonoids, iridoids, and volatile oil comprise of the pharmacological properties. Recently, LJF has also been applied to suppress COVID-19 [13], which poses a substantial threat to public health worldwide. In addition to being used in traditional Chinese medicine, LJF is also used as a food additive in tea, beverage, and wine, which is called “functional food” [14]. Among, chlorogenic acid and luteoloside are the major qualitative markers of LJF recorded in the Chinese pharmacopeia. They are phenolic acid and flavonoid derived from the phenylpropanoid pathway. However, the accumulation and synthesis of metabolites is the result of the interaction of endogenous genes and exogenous environment factors. Therefore, a systematic quality assessment method is needed for the quality control of herbal medicines under salt stress.

Salinity stress promotes the accumulation and synthesis of effective components in LJF, especially chlorogenic acid, and other phenolic components, but the precise mechanisms of the action need further attention [15–17]. Hence, we speculated that salinity might enhance the quality of LJF. Nevertheless, controlling or evaluating the quality of herbal medicine using bioactive compounds is biased, as the complexity and incomplete knowledge of the bioactive compounds. Hence, it is necessary to systemically investigate bioactive compounds and their synthesis in LJF under salt stress to select the optimal growth environment for medicinal quality improvement. The metabolomic approach has been widely applied to comprehensively analyze metabolites and discover biomarkers of salt stress in medicinal plants [18, 19]. LC-MS is regarded as one of the most common methods in metabolomics, which has been widely used to investigate the metabolic profiles of plant materials. Recently, several LC-MS methods are established for qualitative and quantitative analysis of bioactive compounds in traditional Chinese medicine in our laboratory [20–22].

In this study, LJF was treated with varying saline concentrations (0, 100, 200 and 300 mM NaCl) for 35 days. A metabolic profiling method based on High Performance Liquid Chromatography tandem triple time-of-flight mass/mass spectrometer (HPLC-Triple TOF-MS/MS) coupled with multivariate statistical analysis, including principal component analysis (PCA), orthogonal partial least squares-discriminant analysis (OPLS-DA) and hierarchical clustering analysis (HCA) were employed to identify the significant metabolites, discriminate the groups, and screen differential metabolites. Moreover, the metabolic pathways of primary and secondary metabolites were also investigated in LJF to evaluate the effect of salt stress. Our study provides insights into the quality assessment of LJF under salt stress and can be used as a powerful method to improve the medicinal evaluation for the development of LJF.

## Materials and methods

### Chemicals and materials

Methanol and acetonitrile of HPLC grade were purchased from Merck (Damstadt, Germany); ultrapure water was prepared using a Milli-Q purifying system (Millipore, Bedford, MA, USA); standard compounds of chlorogenic acid, cryptochlorogenic acid, neochlorogenic acid, isochlorogenic acid A, isochlorogenic acid B, ferulic acid, caffeic acid, loganin, proline, alanine, serine, leucine, lysine, histidine, arginine, cytidine, uridine, adenosine, and phenylalanine were obtained from Shanghai Yuanye Biotechnology Co. Ltd (Shanghai, China); 1,3‐O‐dicaffeoylquinic acid, isochlorogenic acid C, protocatechuic acid were purchased from Chengdu Prefa Technology Development Co. Ltd (Sichuan, China); quinic acid, rutin, hyperoside, astragalin, isoquercitrin were acquired by the Control of Pharmaceutical and Biological Products (Beijing, China); diosmetin, apigenin, kaempferol, kaempferol‐3‐O-rutinoside, sweroside were purchased from Chengdu Chroma Biotechnology Co. Ltd (Sichuan, China); 4,5‐O-dicaffeoylquinic acid methyl ester, luteolin, luteoloside, rhoifolin, lonicerin, secologanic acid, loganin acid, morroniside were provided by Liangwei Chemical Reagent Co. Ltd (Nanjing, China); secoxyloganin were taken from Nanjing Jingzhu Biotechnology Co. Ltd (Nanjing, China). The purity of all standard compounds was more than 98%.

### Plant and salinity treatments

**Fig 1:**
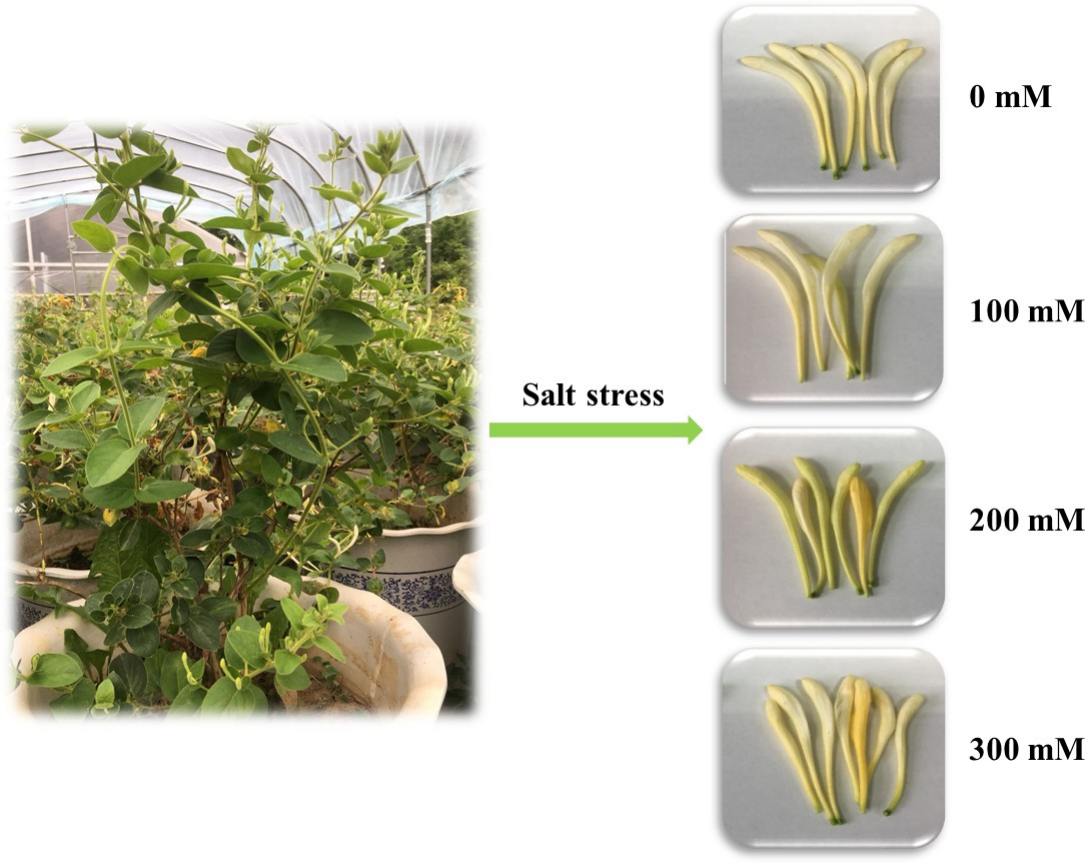
Plant materials. LJF treated with different salt stress conditions.

*L*. japonica was planted in December 2018 at Medicinal Botanical Garden of Nanjing University of Chinese Medicine (north latitude 118°57′1′′, east longitude 32°6′5′′), Nanjing, Jiangsu province, China. The two-year-old main roots of *L*. japonica were transplanted from Henan province, China, which was identified by Professor Xunhong Liu (Department for Authentication of Chinese Medicines, Nanjing University of Chinese Medicine). Then they were planted in plastic plots (50 cm height, 34 cm of top diameter, and 26 cm of bottom diameter). The experiment conditions were similar to the opened-air environment, except a shed blocking off rainwater was installed when the salinity stress began. Four concentrations of salt treatment (0, 100, 200, 300 mM/L NaCl) were conducted since April 24, 2019 and last for 35 days. Design treatments for each concentration in 5 replicates. In order to avoid the osmotic shock, the concentration was increased gradually until meet the set salt concentration. The stress treatment was executed at a rate of 2 L per pot every three days. Then the flower buds were collected from 5 randomly selected plants in May 2019 (Fig 1).

### Sample preparation

The fresh samples (control and salt-treated) were harvested after 35 days of salt stress and then dried in natural conditions. The dried samples of 4 groups were smashed into homogeneous size powder, which was filtered with a 50-mesh screen. Then 1.0 g of the powder was accurately weighed, and extracted using ultra-sonication in 40 mL 70% methanol for 45 min, and then cooled to room temperature; the volatile solution was compensated with 70% methanol and mixed well. The solution was centrifuged at 8050 g for 10 min and filtered through 0.22 nm membrane filters (Jinteng laboratory equipment Co., Ltd., Tianjin, China) before being subjected to HPLC-Triple TOF-MS/MS analysis. Then, the supernatants were stored in a sample bottle at 4°C, and the rest of the freeze-dried powder was stored for other analytical tests.

### HPLC-triple TOF-MS/MS analysis

The separation was performed using the Agilent ZORBAX SB C18 column (Agilent, Palo Alto, CA, USA). The mobile phase was composed of 0.1% aqueous formic acid (A) and acetonitrile with 0.1% formic acid (B) at the flow rate of 0.3 mL/min. 1 μL injection volume and column temperature 35°C. The gradient elution in the following separation program: 0–4 min (2% B); 4–5 min (2–10% B); 5–25 min (10–18% B); 25–29 min (18–25% B); 29–30 min (25–44% B); 30–33 min (44–48% B); 33–38 min (48–72% B); 38–45 min (72–95% B).

The Triple-TOF-MS was operated on AB SCIEX Triple TOF TM 5600 System-MS/MS (AB SCIEX, Framingham, MA, USA) equipped with an electronic spray ionization (ESI) source in positive and negative ion mode. The full scan mass range was set to m/z 100–2000 to acquire TOF-MS data, the scanning range of m/z 50 to 1500 to acquire TOF-MS/MS. The dynamic range enhancement was used throughout the MS experiment to ensure accurate mass measurements. The optimized MS analysis conditions were set as follows: nebulizer gas (GS 1), 55 psi; heater gas (GS 2), 55 psi; curtain gas (CUR), 40 psi; ion spray voltage floating (ISVF), 4500 V; turbo spray temperature (TEM), 550°C; declustering potential, -100 V; collision energy, -40 V.

### Data processing and multivariate statistical analysis

The HPLC-Triple TOF-MS/MS data were processed by MarkerView 1.2.1 software (AB Sciex, USA), the characteristic peaks were inferred through comparing MS/MS fragment ions using PeakView1.2 (AB Sciex, USA), retention time (*tR*) and mass data (m/z) pairs with standard compounds and online resources CNKI, Pubmed, SciFinder, HMDB.

The data from PeakView1.2 and MarkerView were further exported to SIMCA-P software (Version 13.0, Umetrics AB, Umea, Sweden) for multivariate statistical analysis. Unsupervised PCA was performed to elucidate the total metabolic differences among the samples between control and salinity stress groups. The supervised OPLS-DA was carried out multiple times. The ellipses shown in the score plots of PCA and OPLS-DA was defined as a 95% confidence interval for model changes. VIP representing the weighted sum of squares of OPLS-DA analysis was performed. When VIP was great than 1, there was a significant difference in metabolites. Hierarchical clustering analysis of samples based on the significantly changed metabolite was executed by the software of Heml 1.0 Heatmap Illustrator. The column represents 47 metabolites with significant differences, and the row represents samples with different salt stress conditions, respectively.

### Biological pathway analysis

Biological pathway analysis was constructed based on significantly changed metabolites among control and salt stress groups according to MetaboAnalyst 4.0 online (http://www.metaboanalyst.ca/) and KEGG website (http://www.genome.jp/kegg/). Arabidopsis thaliana (thale cress) was selected as a pathway library.

## Results

### Identification of differential metabolites between salt stress and control samples

**Fig 2:**
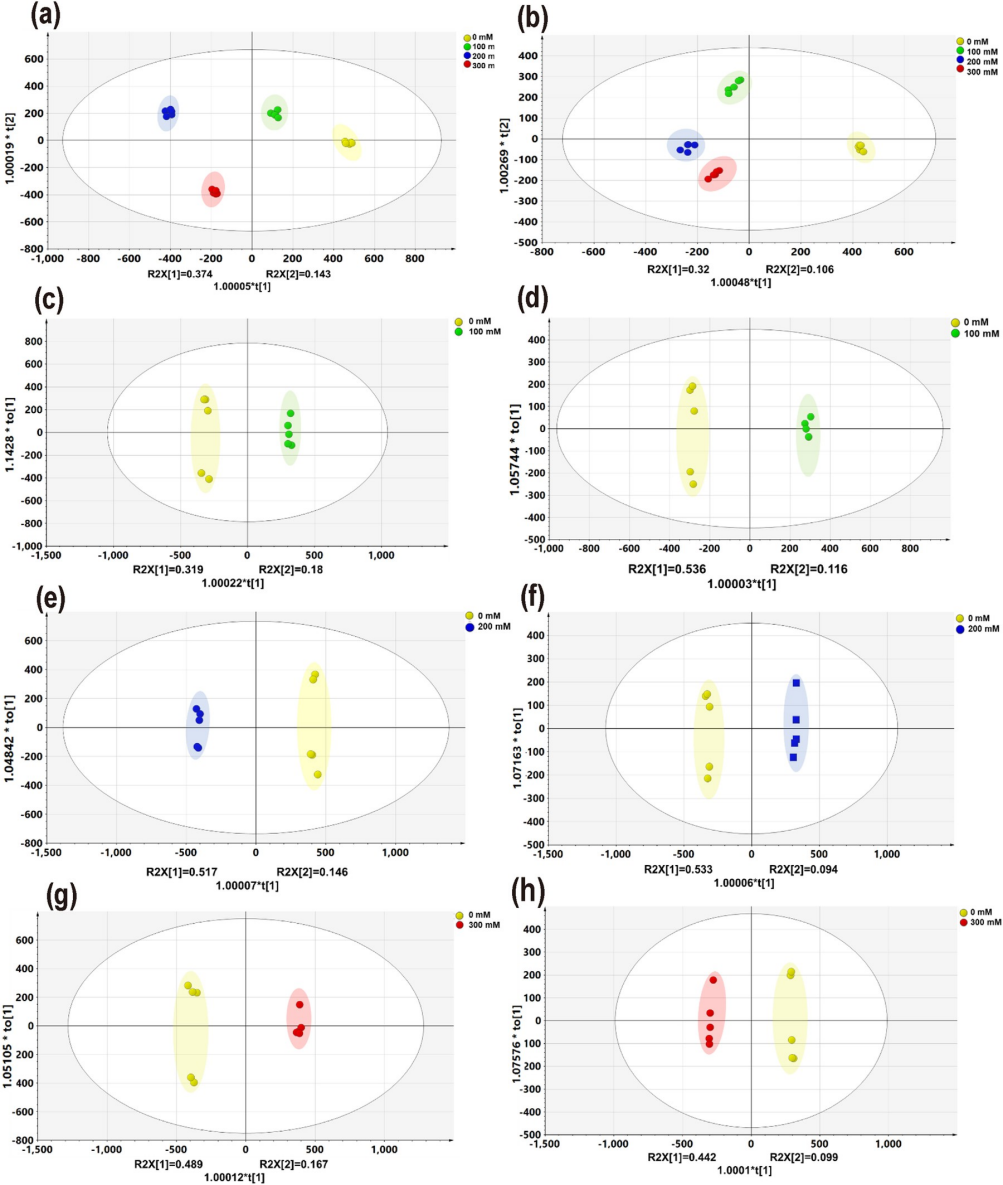
OPLS-DA scores plots of LJF exposed to different salt stress conditions compared with control under negative (a, c, e, and g) and positive (b, d, f, and h) ion modes, respectively. a and b, salt vs control; c and d, 100 mM salt vs control; e and f 200 mM salt vs control; g and h 300 mM salt vs control. 95% confidence regions are displayed for each treatment.

**Fig 3:**
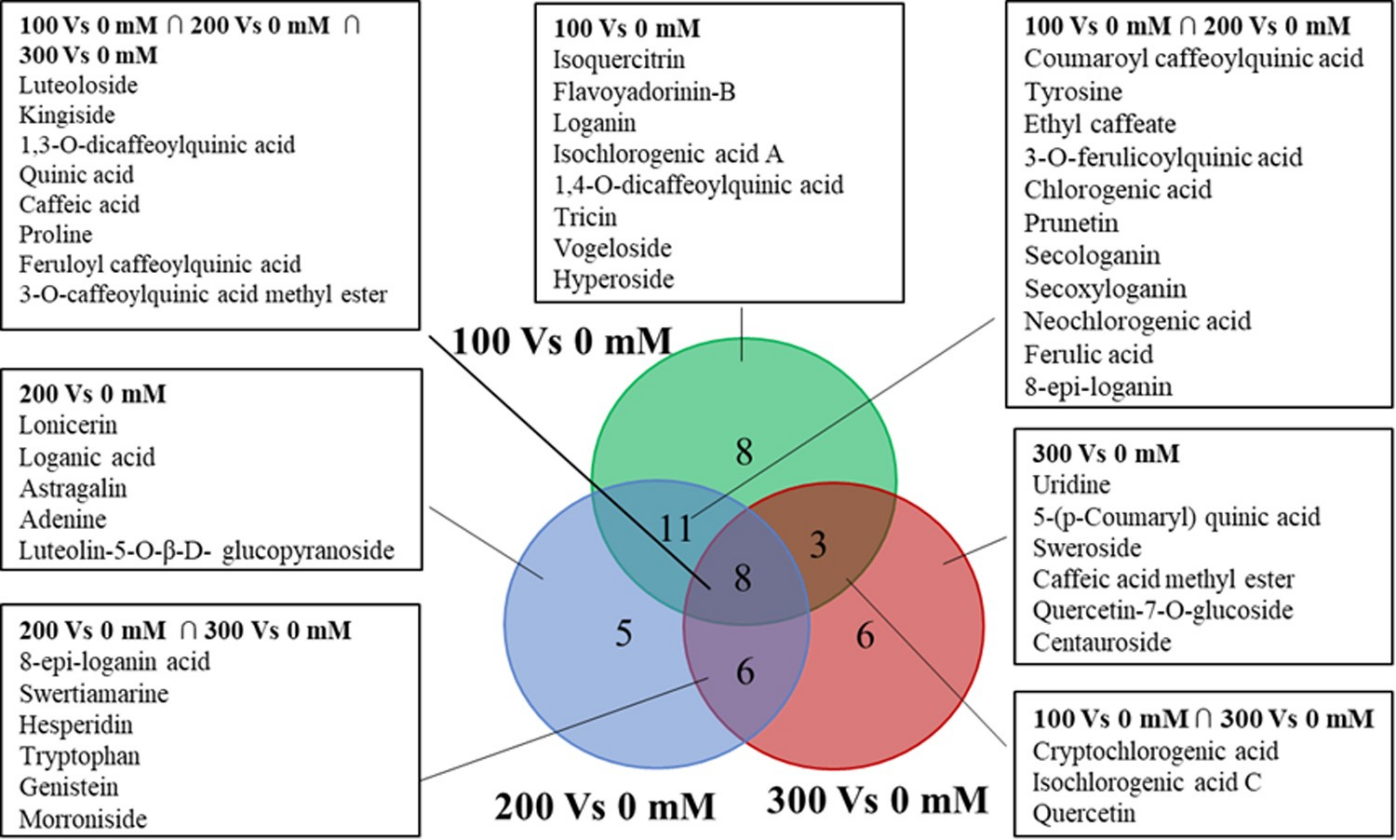
Venn diagram analysis of differentially metabolites under salt treatment compared to the control group. In the Venn diagram, overlapping areas indicated that metabolites are shared between corresponding groups, while the remaining areas showed specific metabolites.

To investigate the metabolites changes of LJF under different salt stresses (0, 100, 200, 300 mM NaCl), a metabolomic method based on HPLC-Triple TOF-MS coupled with multivariate statistical analysis was applied. The preliminary experiments of our research had found that a total of 300 compounds were detected in LJF by HPLC-Triple TOF-MS (S1 Fig), among them, 79 compounds were identified and characterized by comparisons of retention times, mass-to-charge ratio and secondary mass spectrum with authentic standards. The list of 79 compounds including 24 phenolic acids, 23 flavonoids, 15 iridoids, 12 Amino acids, 5 nucleosides was provided as S1 Table and 47 differential metabolites were screened. The PCA could be categorized into 4 groups of LJF according to the different salt-treated (S2 Fig). Supervised OPLS-DA analysis of the control and salt stress groups showed that the control group could be completely separated from salt stress groups in positive and negative mode, respectively (Fig 2A and 2B); and 47 differential metabolites were screened from LJF under different salt stress conditions (Fig 3). Low salt stress group (100 mM NaCl) appeared to be better than other samples in terms of relative contents (peak areas) of the wide variety of bioactive components, indicating that LJF treated with 100 mM NaCl had the best quality. Besides, the phenylpropanoid pathway, monoterpenoid biosynthesis, glycolysis, TCA cycle, and alkaloid biosynthesis were disturbed in all salt-stress LJF.

### Metabolite profile changes under low salt-treated

The relative contents of caffeoylquinic acid derivatives including chlorogenic acid, neochlorogenic acid, 1,3-O-dicaffeoylquinic acid, 1,4-O-dicaffeoylquinic acid, isochlorogenic acid A showed a significant accumulation under control group and low salt-treated group. In more detail, the relative contents of quinic acid (1.15-fold), 1,3-O-dicaffeoylquinic acid (2.1-fold), 1,4-O-dicaffeoylquinic acid (2.21-fold), chlorogenic acid (1.3-fold), and neochlorogenic acid (1.0-fold) were increased in LJF under low salt-treated samples. And the relative contents of flavonoid changes in LJF were also obviously different. Such as luteoloside, quercetin, luteolin-5-O-β-D-glucopyranoside, lonicerin, flavoyadorinin-B went up 1.28, 1.78, 0.95, 1.07, 1.15-folds under low salt-treated samples. In addition to phenolics and flavonoids, iridoids are also the bioactive components in LJF. It is noted that loganin (1.81-fold), swertiamarine (1.04-fold), vogeloside (3.87-fold), and secologanin (1.05-fold) also showed an increasing trend at low salt-treated samples. Our study showed that low salt dose increased the level of metabolites, derived from the shikimate-phenylpropanoid pathway were higher in LJF under 100 mM than 0, 200, 300 mM; such as quinic acid, 1,3-O-dicaffeoylquinic acid, chlorogenic acid, luteoloside, quercetin, etc. Follow up studies would validate the role of the described metabolites as markers of the stress response. The OPLS-DA analysis of the control group and low salt-treated group showed that the control group could be completely separated from the salt stress group in positive and negative mode, respectively (Fig 2C and 2D). And the differential compounds between every two groups were screened by combining variable importance in the projection score (VIP) greater than 1, the value of VIP > 1 were showed in S3A Fig.

### Metabolite profile changes under medium salt-treated

In addition to the changes observed at 100 mM NaCl, samples with medium salt-treated also induced caffeic acid (2.02-fold), 1,3-O-dicaffeoylquinic acid (1.15-fold), chlorogenic acid (1.17-fold), 1,4-O-dicaffeoylquinic acid (0.52-fold) compared with the control group; and the key components of flavonoid, especially flavone and flavanol biosynthesis, luteolin-5-O-β-D-glucopyranoside (2.73-fold), lonicerin (3.01-fold), flavoyadorinin-B (2.29-fold), the relative contents rose more at 200 Mm NaCl; besides, hyperoside (1.22-fold), genistein (1.08) have also increased. However, the synthesis of quercetin was reduced under medium salt stress; it might indicate quercetin was used to synthesis other flavonoids to defense salt stress. Through OPLS-DA analysis, the control group and low salt-treated group could be completely separated in positive and negative mode, respectively (Fig 2E and 2F); then the differential compounds between every two groups were screened by combining variable importance in the projection score (VIP) greater than 1, and the value of VIP > 1 were showed in S3B Fig.

### Metabolite profile changes under high salt-treated

The changes in the metabolic level of LJF stressed by 300 mM NaCl compared to the control group were shown in Fig 3. It is noteworthy that shikimate-phenylpropanoid pathway metabolites (e.g. quinic acid (1.23-fold), caffeic acid (1.18-fold), ferulic acid (0.92-fold), isochlorogenic acid A (1.71-fold), isochlorogenic acid C (0.49-fold), luteolin-5-O-β-D-glucopyranoside (4.25-fold), lonicerin (3.67-fold), flavoyadorinin-B (0.81-fold), isoquercitrin (1.05-fold), and astragalin (1.71-fold)) were prominently decreased compared to the control group. And the iridoids such as morroniside (1.08-fold), swertiamarine (1.01-fold), loganic acid (0.77-fold) showed a downward trend as well as phenolic acid and flavonoid at high salt-treated samples, while, centauroside (1.89-fold), secologanin (1.49-fold) were increased. In the past decades, more than 30 iridoids have been reported from *L*. japonica and many published studies have shown that iridoid is an important class of plant defensive components found in more than 50 plant families [23–25]. In this study, 15 iridoids were identified from LJF treated with different salt stress. Such as loganin, kingiside, secoxyloganin, sweroside, loganin acid, morroniside, vogeloside, and swertiamarine. By OPLS-DA analysis, the control group and high salt-treated group could be completely separated in positive and negative mode, respectively (Fig 2G and 2H); then the differential metabolites were screened and showed in S3C Fig. To our knowledge, there is no information on the changes of iridoids in LJF under different salt stress. The results provided detailed information for the quantification of iridoids in LJF.

### Multivariate statistical analysis

**Fig 4:**
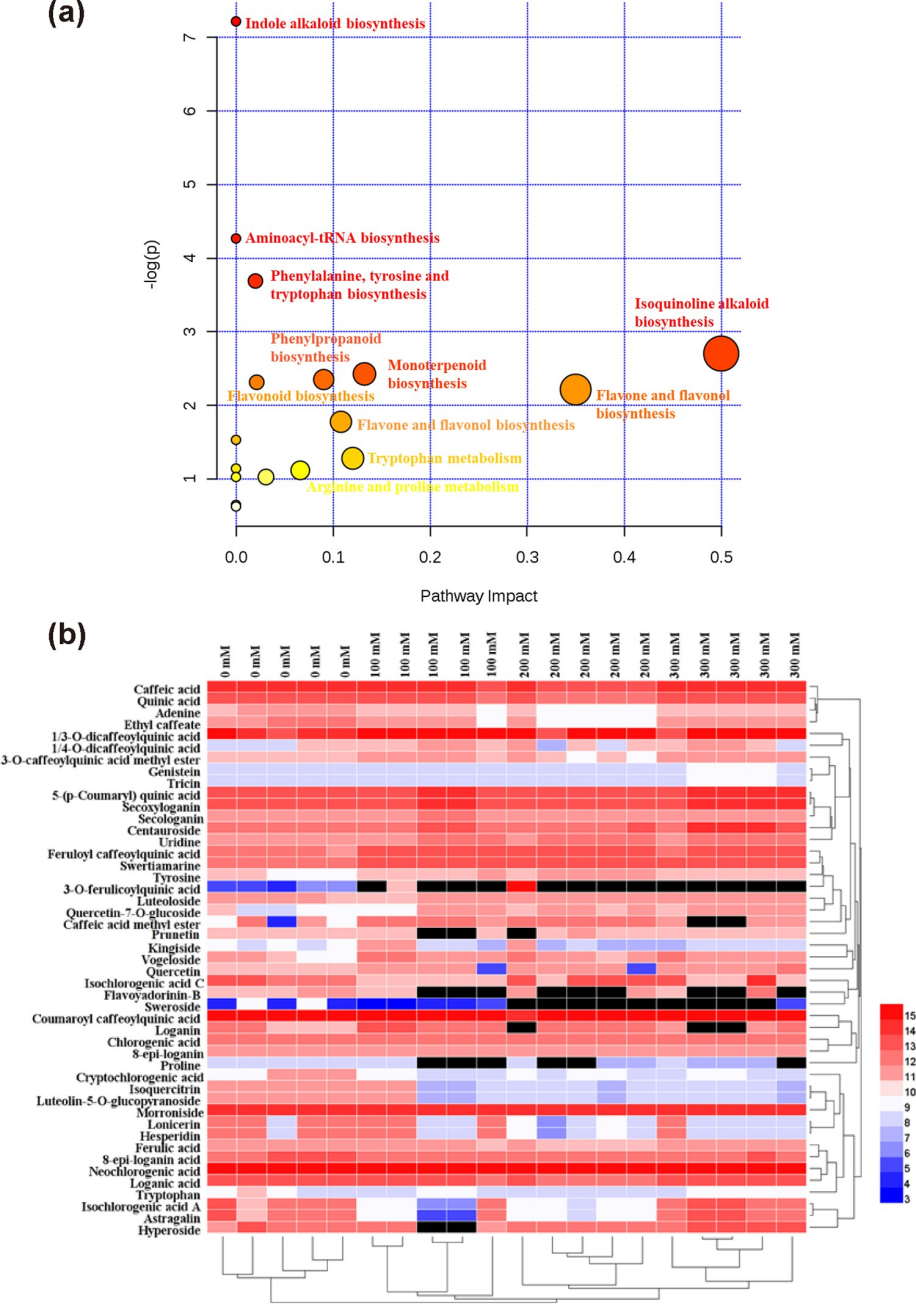
The significantly changed biological pathway under salt stress (a); Hierarchical clustering analysis of differentially metabolites in LJF under different salt treatment compared to the control group (b). The colors from blue to red graphically represented the relative content of metabolites from low to high.

Multivariate statistical analysis was applied to reduce the data complexity and better discovery for the potential differential metabolites. The unsupervised PCA was performed with principal components 1 and principal components 2 to get a general description of the variance of metabolites. As shown in S2 Fig of the PCA score plot, four groups of samples with different salinities were completely separated. The supervised OPLS-DA was carried out to better distinguish the four salinity samples and differential metabolites. The validity of the OPLS-DA model was assessed with permutation tests (S4 Fig), indicating that there was no overfitting phenomenon in this model. The model parameters were R2X = 0.812, R2Y = 0.995, and Q2 = 0.906 in Fig 2A; R2X = 0.787, R2Y = 0.977, and Q2 = 0.832 in Fig 2B. In both cases, these results indicated that the model was stable with strong predictable ability under the positive and negative ion mode, the control group and the different salt stress groups could also be completely separated. Furthermore, Fig 2A and 2B showed that plants treated with different salt stress were completely separated from control. The differential metabolites were identified and discovered by the components of *L*. japonica, standards, relevant literature, and variable importance in the projection (VIP) score; the compound was regarded as a potential marker when its VIP-value was greater than 1 [26]. Then, OPLS-DA analysis was conducted in each group at 100 mM vs control (Fig 2C and 2D), 200 mM vs control (Fig 2E and 2F), 300 mM vs control (Fig 2G and 2H), respectively, to distinguish the differences of metabolites under different salt stress. The metabolic pathways of 47 differential metabolites based on the control group and different salt-treated samples were displayed in Fig 4A, these changes indicated salt stress had a wide range of effect in LJF; Clear differentiation was observed in the heat map (Fig 4B and S2 Table). Besides, the similarity evaluation of clustering was consistent with the OPLS-DA analysis, both were higher in the content of metabolites at 100 mM NaCl. These results suggested the quality of LJF under 100 mM salt stress was better than the other three groups.

## Discussion

### Metabolite profile changes under salt stress

The variation in metabolites of LJF is significantly different between the control and salt-treated samples. Phenolics compound are the most used dietary antioxidants, most of them were derived from the phenylpropanoid pathway. Especially phenolic acid and flavonoid are major components in LJF; Among them, luteoloside and chlorogenic acid are the quantitative component in LJF. Therefore, it is necessary to study how luteoloside, chlorogenic acid and other bioactive components distribute in plant under salt stress. It is reported that the elevated level of chlorogenic acid could enhance oxidative damage tolerance [27]; and caffeic acid is not only rapidly absorbed and maintained metabolically stable but also has the same antioxidant capacity as chlorogenic acid [28, 29]. In addition, these ingredients also exhibit other pharmacological activities such as anti-inflammatory, antiviral, antibacterial, cytoprotective, immunomodulatory, antihyperglycemic, and anticancer. And we found that the contents of phenolic acid were much higher under low salt stress compared to other groups. Flavonoids are a class of compounds with 2-phenylchromone structure. It plays an important role in plant growth, development, flowering, fruiting, and antibacterial, antiviral, and antioxidant aspects. In recent years, much effort has been performed to elucidate the flavonoid biosynthetic pathway from a molecular genetic point of view [30]; and several breakthroughs have been made in the flavonoid pathway. Although Arabidopsis thaliana is helpful to analyze the regulation pathway of flavonoids, it is not exactly applicable to non-model plants. Hence, more research needs to be conducted, especially in medicinal plants under salt stress. In this study, the quality marker of chlorogenic acid and luteoloside in LJF increase 1.3, 1.28-fold under salt stress, respectively. This results further support the reported in the literature [16]. And that's consistent with our hypothesis. Besides, primary metabolites not only constitute essential nutrients for plant growth, but also protect cellular membranes integrality under salt stress. In the study, the contents of proline, serine, and lysine all significantly changed under salt stress. And proline, which is an osmoprotectant, be screened out as a differential marker under salt stress; This may be related to the salt tolerance of LJF. In addition, the molecular mechanism that the accumulation of secondary metabolites in LJF under low salt stress, which will be further analyzed combining with the data of proteomics, transcriptomics, and genomics.

### Global visualization of pathways changes and proposed phenolic acid, flavonoid and iridoid biosynthesis of LJF exposed to salt stress

**Fig 5:**
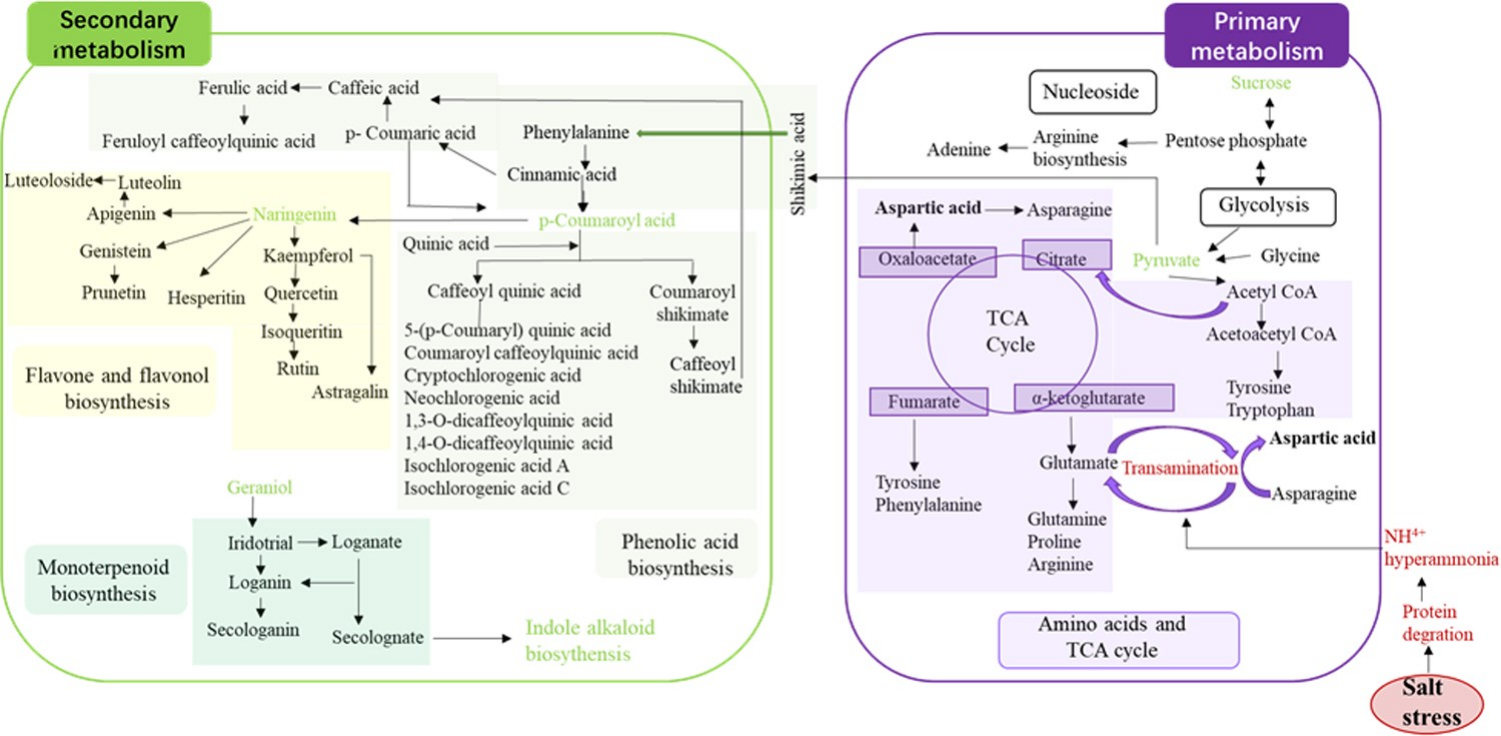
Metabolic pathways of differential metabolites in LJF under salt stress. Metabolic pathways of phenolic acids and its interactions with other pathways in LJF under salt stress.

The chemical interactions between plants and the environment are mainly mediated by the biosynthesis of secondary metabolites, and much of these metabolites are derivatives of primary metabolites produced by plants [31]. Primary metabolite citric acid is a key metabolic component of the tricarboxylic acid cycle (TCA cycle), generating energy and providing adaptive flexibility for adverse environments. Most secondary metabolite biosynthesis starts from basic pathways, such as glycolysis or shikimic acid pathways [32]. Shikimic acid is a precursor for the aromatic amino acids, alkaloids, tannins, and lignin. According to the type of secondary metabolites in plants, they can be divided into three major classes of biosynthetic pathways including nitrogen-containing compounds (cyanogenic glycosides, alkaloids, and glucosinolates), phenolic compounds (flavonoids and phenylpropane), and terpenes (isoprenoids). In this study, we investigate some metabolic pathways including phenolic acid, flavonoid, iridoid biosynthesis, glycolysis, TCA cycle, and alkaloid, then selecting the representative pathways for more detailed analysis. The biosynthesis OF metabolite is subject to dynamic regulation by metabolic feedback and environmental factors. Therefore, to future research how these metabolites distributed under oxidative stress is important. Specifically, chlorogenic acid and luteoloside, which is the chemical markers for evaluating the quality of LJF in Chinese Pharmacopoeia (2015 edition) [33]. Chlorogenic acid is one of the most widely known derivatives of hydroxycinnamic acid in the plant kingdom, and numerous reports on the biosynthesis of chlorogenic acid have been published [34]. However, the biosynthetic pathway for generating chlorogenic acid is still controversial; hence, more studies remain to be carried out. The phenolic acid, flavonoid, and iridoid biosynthesis of LJF under salt stress were inferred in Fig 5. Generally, the biosynthesis of phenolic compounds is believed to be primarily regulated at the transcriptional level; the above synthetic pathway provided in the reference for phenolic acid synthesis facilitated a better understanding of its transcriptional regulation. Meanwhile, it also provides new ideas for the selection of regulatory genes for metabolic engineering under salt stress. And the published studies also focus on luteoloside and flavonoid biosynthesis [30], the subgroup pathway including isoflavinoid, flavone, and flavonol biosynthesis also should be attentioned. Besides, the pathways of iridoid metabolites were also mentioned in the study. In the subsequent, we will utilize multivariate omics data [35] to better understanding the molecular changes/mechanism of LJF under salt stress, the prognostic molecular pathways will be analyzed by integrating genomic, transcriptomic and proteomic. Overall, an in-depth study of the molecular mechanism and network regulation of bioactive constituents synthesis in medicinal plants under salt stress will be an crucial research directions.

## Conclusions

In summary, the present study demonstrated that the combination of LC-MS-based metabolomics and multivariate statistical analysis was an effective approach to distinguish and qualify LJF under different salt stress. A total of 79 compounds were identified in LJF treated with different salt stresses, 47 of them were differential compounds. Differential metabolites indicated that secondary metabolites, which were close to the antioxidant capacity, such as phenolic acid, flavonoid, and iridoid were affected in all LJF samples exposed to salt stress. Among these metabolites, significantly increases were observed at 100 mM NaCl treatment compared to the control group, while the relative contents (peak areas) of metabolites were decreased under 300 mM, indicating that low salt-treated group appeared to be better in terms of relative contents of the wide variety of bioactive components and excess salts in the soil are harmful to the development and quality of plants. Meanwhile, the biosynthesis of phenolic acid, flavonoid, and iridoid were influenced in all LJF samples under salt stress. These metabolomic changes exposed to salt stress were first reported on LJF and clearly involved in a network. Our study provides insights into the quality assessment of LJF under salt stress and can be used as powerful complementation to improve the medicinal value for the development of LJF.

## Supporting information

S1 Fig Representative UFLC-Triple TOF MS/MS base peak chromatogram of LJF sample under negative (a) and positive (b) ion modes, respectively.
(DOCX)

S2 Fig PCA scores plot of LJF induced by different salt stress under positive (a) and negative (b) ion modes, respectively.
(DOCX)

S3 Fig VIP score plot derived from OPLS-DA of LJF induced by low (a, 100 mM), medium (b, 200 mM) and high (c, 300 mM) concentration of salt compared to the control.
(DOCX)

S4 Fig Permutation test with 200 permutations of model (in the positive ion mode (a), in the negative ion mode (b)).
(DOCX)

S1 Table Identification of 82 metabolites in LJF by UFLC-Triple TOF-MS/MS. (DOCX)

S2 Table The relative contents of differentially metabolites in LJF under different salt. (DOCX)
