# Supplementary material for: Metabolomics characterizes the metabolic changes of Lonicerae Japonicae Flos under different salt stresses
Source: PLoS One. 2020 Dec 1;15(12):e0243111. doi: 10.1371/journal.pone.0243111 (PMC7707481; doi:10.1371/journal.pone.0243111)
Supplement: S1 Fig — Representative UFLC-Triple TOF MS/MS base peak chromatogram of LJF sample under negative (a) and positive (b) ion modes, respectively. (DOCX) [file pone.0243111.s001.docx]

**
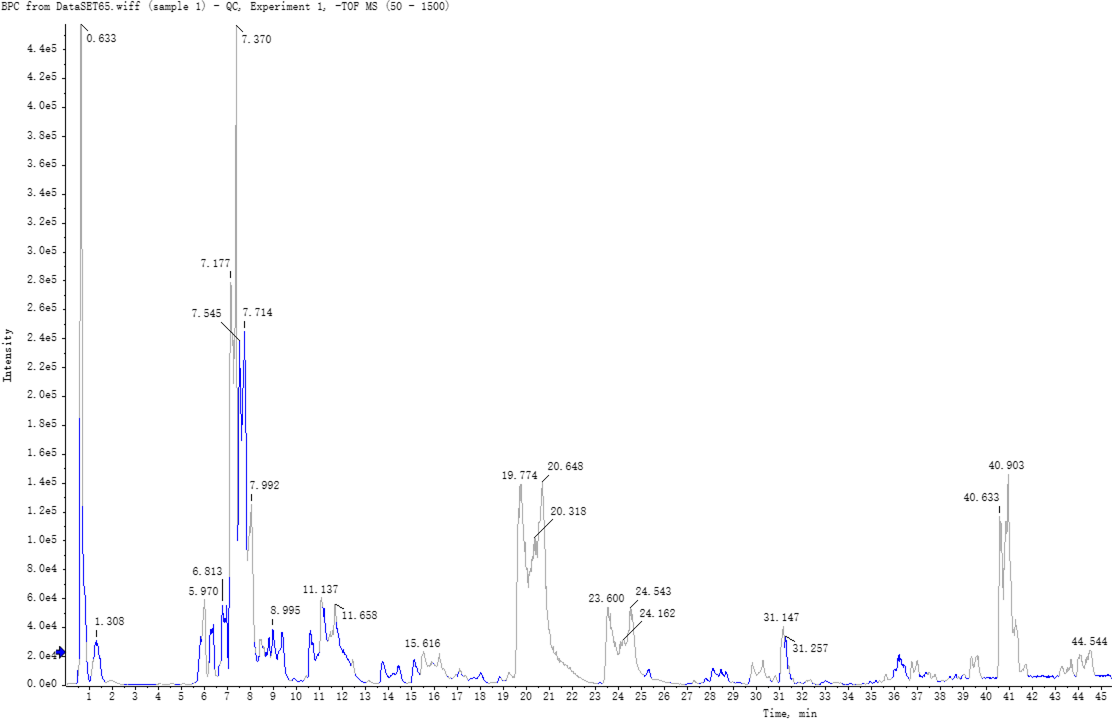
**

**(a)**

**
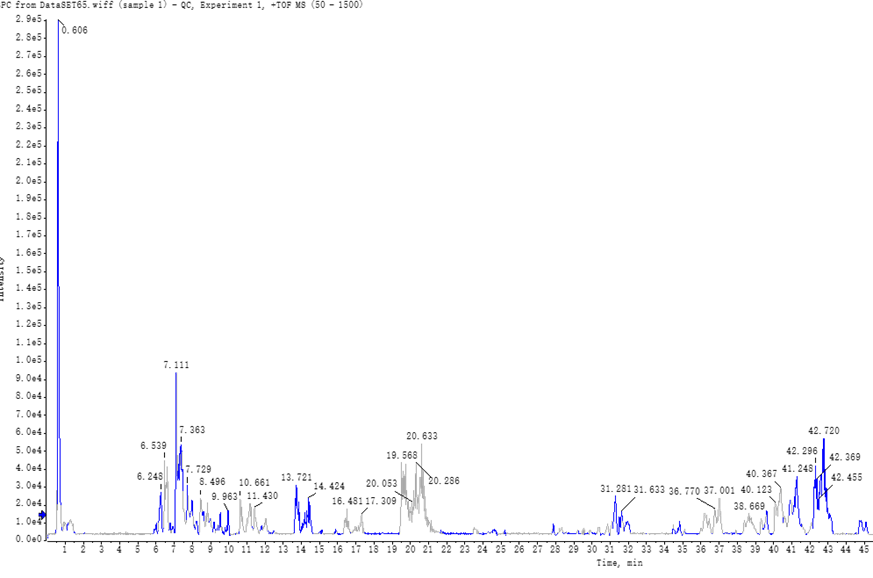
**

**(b)**

**S1 Fig** Representative UFLC-Triple TOF MS/MS base peak chromatogram of LJF sample under negative (a) and positive (b) ion modes, respectively.
