# Supplementary material for: Metabolomics characterizes the metabolic changes of Lonicerae Japonicae Flos under different salt stresses
Source: PLoS One. 2020 Dec 1;15(12):e0243111. doi: 10.1371/journal.pone.0243111 (PMC7707481; doi:10.1371/journal.pone.0243111)
Supplement: S2 Fig — PCA scores plot of LJF induced by different salt stress under positive (a) and negative (b) ion modes, respectively. (DOCX) [file pone.0243111.s002.docx]

**(a)
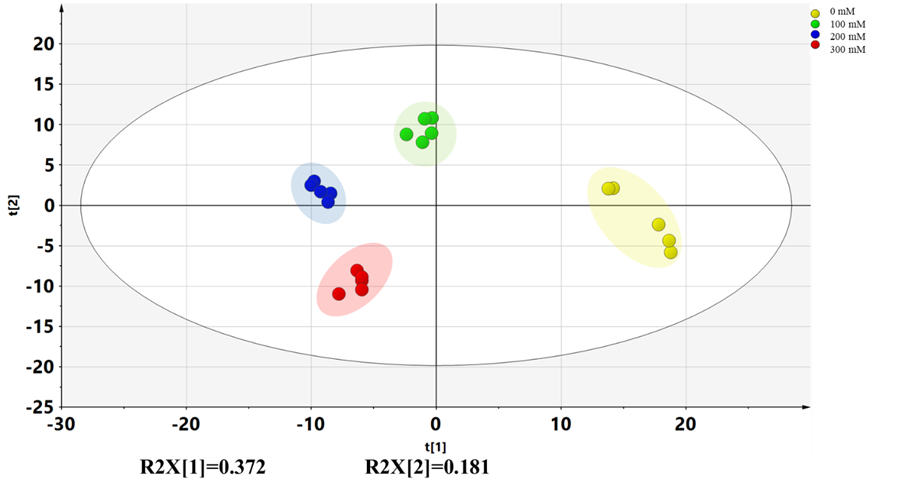

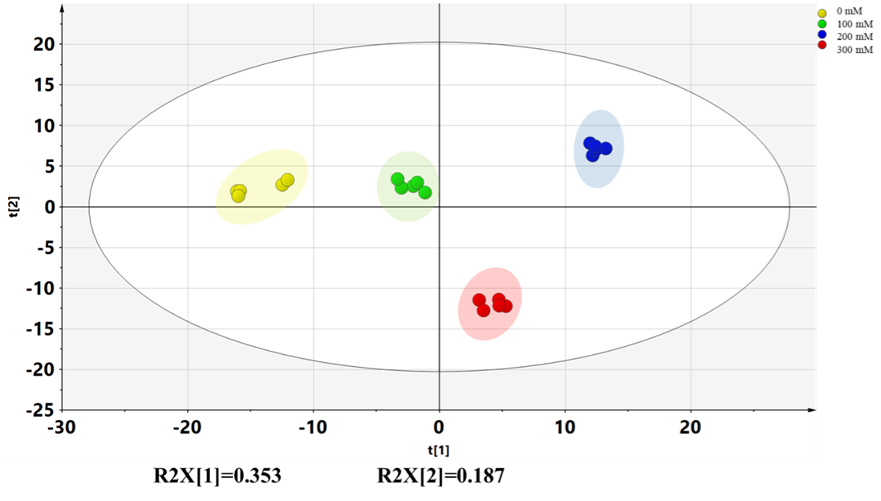
(b)**

**S2 Fig** PCA scores plot of LJF induced by different salt stress under positive (a) and negative (b) ion modes, respectively.
