# Supplementary material for: Metabolomics characterizes the metabolic changes of Lonicerae Japonicae Flos under different salt stresses
Source: PLoS One. 2020 Dec 1;15(12):e0243111. doi: 10.1371/journal.pone.0243111 (PMC7707481; doi:10.1371/journal.pone.0243111)
Supplement: S3 Fig — VIP score plot derived from OPLS-DA of LJF induced by low (a, 100 mM), medium (b, 200 mM) and high (c, 300 mM) concentration of salt compared to the control. (DOCX) [file pone.0243111.s003.docx]

| **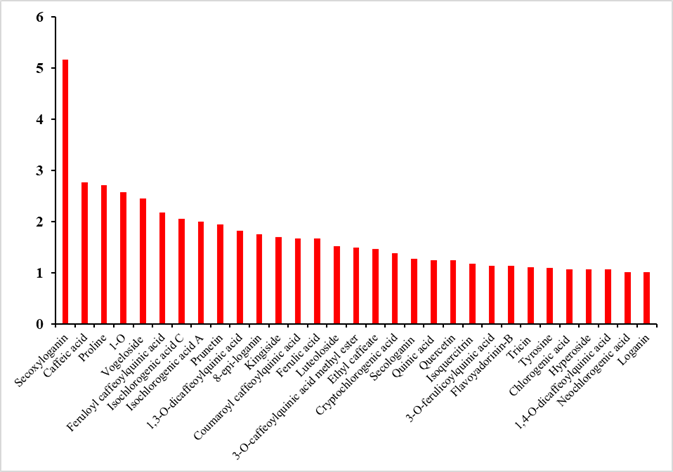**  **(a)** | **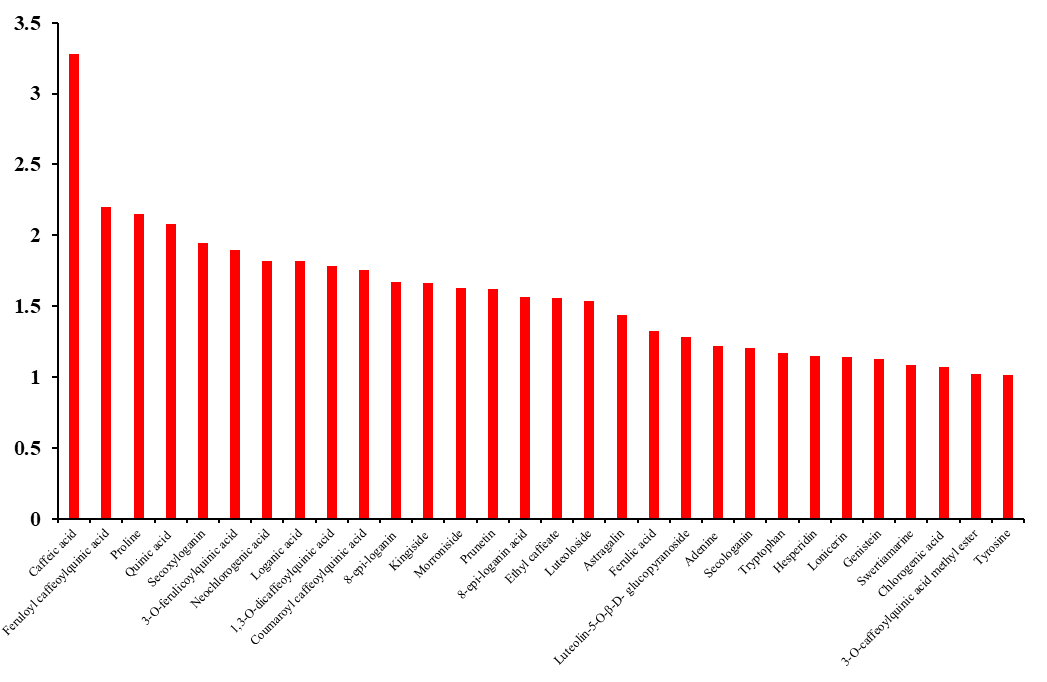**  **(b)** |
| --- | --- |
| **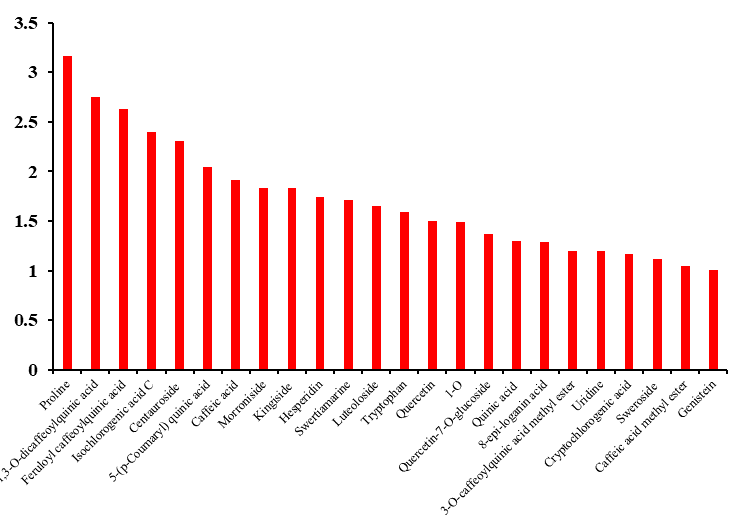**  **(c)** |  |

**S3 Fig** VIP score plot derived from OPLS-DA of LJF induced by low (a, 100 mM), medium (b, 200 mM) and high (c, 300 mM) concentration of salt compared to the control.
