# Supplementary material for: Metabolomics characterizes the metabolic changes of Lonicerae Japonicae Flos under different salt stresses
Source: PLoS One. 2020 Dec 1;15(12):e0243111. doi: 10.1371/journal.pone.0243111 (PMC7707481; doi:10.1371/journal.pone.0243111)
Supplement: S4 Fig — Permutation test with 200 permutations of model (in the positive ion mode (a), in the negative ion mode (b)). (DOCX) [file pone.0243111.s004.docx]

| **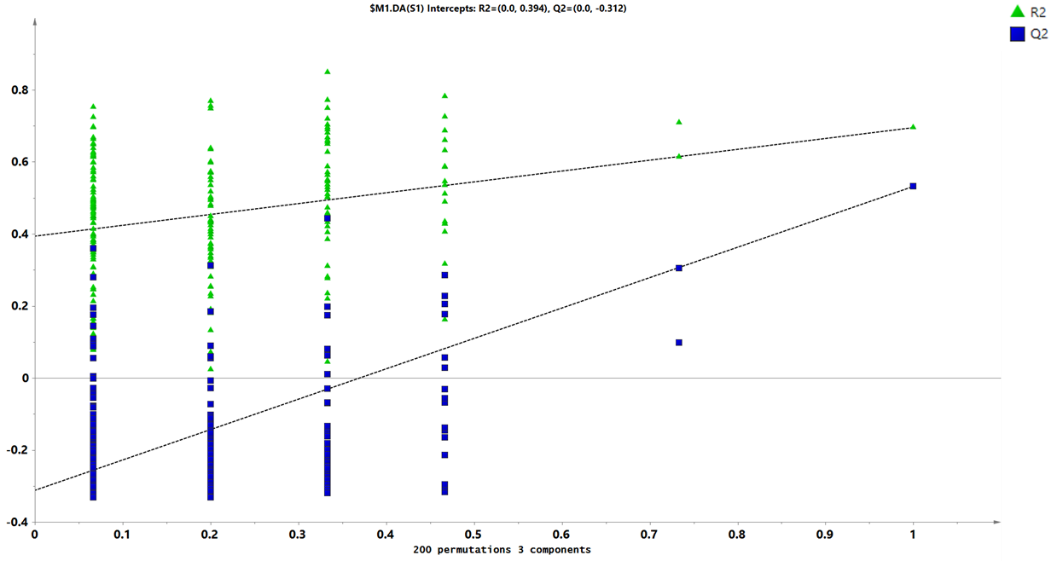**  **(a)** | **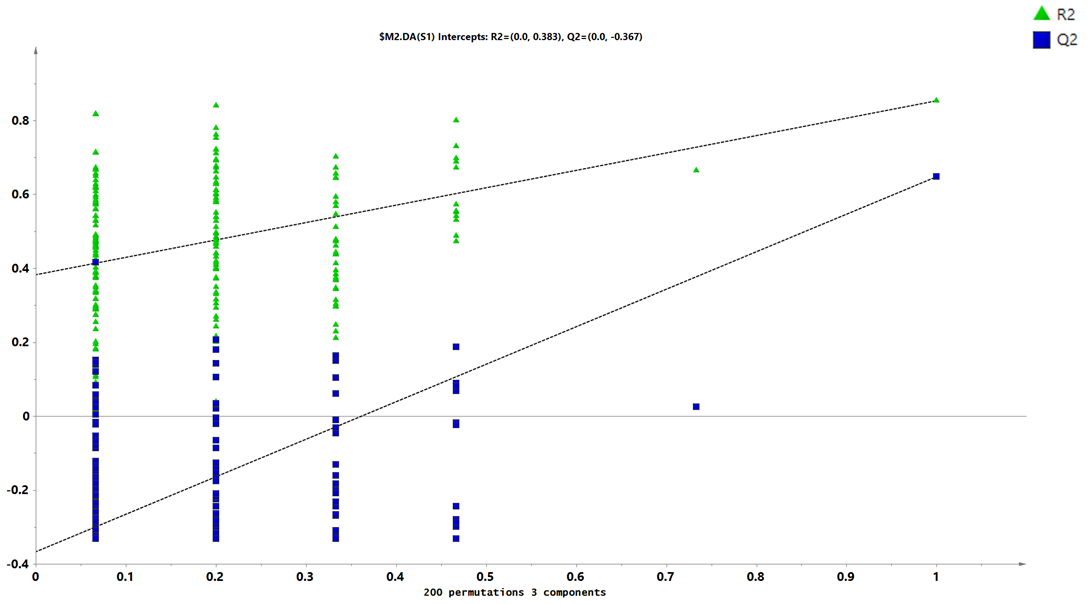**  **(b)** |
| --- | --- |

**S4 Fig** Permutation test with 200 permutations of model (in the positive ion mode (a), in the negative ion mode (b))
