# Supplementary material for: Metabolomics characterizes the metabolic changes of Lonicerae Japonicae Flos under different salt stresses
Source: PLoS One. 2020 Dec 1;15(12):e0243111. doi: 10.1371/journal.pone.0243111 (PMC7707481; doi:10.1371/journal.pone.0243111)
Supplement: S1 Table — (DOCX) [file pone.0243111.s005.docx]

**S1** **Table** Identification of 82 metabolites in LJF by UFLC-Triple TOF-MS/MS

| **No.** | **Identification compounds** | **RT(min)** | **Molecular formula** | **Proposal ions** | **ppm** | **MS/MS fragment** | **Classification** |
| --- | --- | --- | --- | --- | --- | --- | --- |
| 1 | Proline* | 0.65 | C_5_H_9_NO_2_ | [M-H]^-^ | 1.1 | 114.9905[M-H]^-^,56.9982[M-H-C_3_H_3_O_2_-CH_3_]^-^ | Amino acid |
| 2 | Alanine* | 0.56 | C3H7NO2 | [M-H]^-^ | 2.4 | 88.0404[M-H]^-^ | Amino acid |
| 3 | Serine* | 0.55 | C3H7NO3 | [M-H]^-^ | 1.7 | 104.0371[M-H]^-^,74.0276[M-H- CH_3_-CH_3_] | Amino acid |
| 4 | Leucine* | 2.10 | C_6_H_13_NO_2_ | [M-H]^-^ | 4.1 | 130.0878[M-H]^-^ | Amino acid |
| 5 | Lysine* | 0.53 | C6H14N2O2 | [M-H]^-^ | 3.6 | 145.0627[M-H]^-^, 128.0353[M-H-NH_3_]^-^ | Amino acid |
| 6 | Histidine* | 0.54 | C6H9N3O2 | [M-H]^-^ | 3.7 | 154.9478[M-H]^-^ | Amino acid |
| 7 | Arginine* | 0.51 | C6H14N4O2 | [M-H]^-^ | 3.7 | 173.0453[M-H]^-^, 111.0092[M-H-3CH_3_- NH_3_]^-^ | Amino acid |
| 8 | Cytidine | 0.51 | C9H13N3O5 | [M-H]^-^ | 3.7 | 242.9393[M-H]^-^,158.9788,114.9895 | Nucleoside |
| 9 | Uridine* | 1.56 | C9H12N2O6 | [M-H]^-^ | -1.2 | 243.06614[M-H]^-^,143.0710 | Nucleoside |
| 10 | Tyrosine | 1.58 | C9H11NO3 | [M-H]^-^ | 0.5 | 180.0668[M-H]^-^,163.0552 | Amino acid |
| 11 | Tryptophan | 6.3 | C11H12N2O2 | [M-H]^-^ | 3.7 | 203.0919[M-H]^-^ | Amino acid |
| 12 | Guanosine | 2.72 | C10H13N5O5 | [M-H]^-^ | -2.9 | 282.0843[M-H]^-^,15.0421,133.0156 | Nucleoside |
| 13 | Adenine* | 0.88 | C5H5N5 | [M-H]^-^ | 3.8 | 134.0502[M-H]^-^,116.0064,107.0367 | Nucleoside |
| 14 | Adenosine | 0.58 | C10H13N5O4 | [M-H]^-^ | -4.7 | 266.0895 [M-H]^-^ | Nucleoside |
| 15 | Quinic acid* | 0.60 | C_7_H_12_O_6_ | [M-H]^-^ | 0.2 | 191.05615^-^,173,154 | Phenolic acid |
| 16 | Valine* | 0.95 | C_5_H_11_NO_2_ | [M-H]^-^ | 5.4 | 101.0608[M-H-NH_3_]^-^,85.0295[M-H-NH_3_-CH_4_]^-^,57.0710[M-H-C_2_H_4_O_2_]^-^ | Amino acid |
| 17 | Isoleucine* | 2.24 | C_6_H_13_NO_2_ | [M-H]^-^ | 3.8 | 130.874[M-H]^-^,98.0611[M-H_2_O-CH_3_]^-^ | Amino acid |
| 18 | Phenylalanine* | 3.50 | C_9_H_11_NO_2_ | [M-H]^-^ | 2.4 | 146.0611[M-H-H_2_O]^-^,103.0553[M-H-COO-NH_3_]^-^, 77.0397[M-H-COO-NH_3_-C_2_H_2_]^-^ | Amino acid |
| 19 | Protocatechuic acid* | 4.21 | C_7_H_6_O_4_ | [M-H]^-^ | 2.4 | 109.0295[M-H-COO]^-^, 91.0189[M-H-COO-H_2_O]^-^ | Phenolic acid |
| 20 | 8-epi-loganin acid | 6.09 | C_16_H_24_O_10_ | [M-H]^-^ | 3.5 | 213.0759[M-H-Glc]^-^,167.0447[M-H-Glc-H_2_O-CO]^-^ | Iridoid |
| 21 | 1-O-caffeoylquinic acid* | 6.40 | C_16_H_18_O_9_ | [M-H]^-^ | 3.1 | 191.0569[M-H-CA]^-^,179.0351,135.0456 | Phenolic acid |
| 22 | Chlorogenic acid* | 7.1 | C_16_H_18_O_9_ | [M-H]^-^ | -1.1 | 191.0568[M-H-CA]^-^,127.0398[[M-H-CA-2H_2_O-CO]^-^ | Phenolic acid |
| 23 | Morroniside* | 7.49 | C_17_H_26_O_11_ | [M+HCOO]^-^ | 4.1 | 451.1476[M-H+HCOOH]^-^,243.0899[M-H-Glc]^-^,191.0573,119.0368,105.0323,101.0263 | Iridoid |
| 24 | Loganic acid* | 7.1 | C_16_H_24_O_10_ | [M-H]^-^ | 3.8 | 329.1492[M-H-H_2_O-CO]^-^,213.0759[M-H-Glc]^-^,169.0871[M-H-Glc-CO_2_]^-^,151.0765[M-H-Glc-CO_2_-H_2_O]^-^ | Iridoid |
| 25 | Neochlorogenic acid* | 7.32 | C_16_H_18_O_9_ | [M-H]^-^ | 3.1 | 191.0569[M-H-CA]^-^,179.0351,135.0456 | Phenolic acid |
| 26 | Cryptochlorogenic acid* | 7.9 | C_16_H_18_O_9_ | [M-H]^-^ | -1.1 | 191.0568[M-H-CA]^-^,127.0398[M-H-CA-2H_2_O-CO]^-^ | Phenolic acid |
| 27 | Caffeic acid* | 8.1 | C_9_H_8_O_4_ | [M-H]^-^ | 2.9 | 135.0460[M-H-CO_2_]^-^ | Phenolic acid |
| 28 | Swertiamarin | 8.37 | C_16_H_22_O_10_ | [M-H]^-^ | 2.8 | 193.0523[M-H-Glc-H_2_O]^-^,149.0617[M-H-Glc-H_2_O-CO_2_]^-^, 119.0356,101.0250 | Iridoid |
| 29 | Secologanic acid* | 8.37 | C_16_H_22_O_10_ | [M-H]^-^ | 2.9 | 193.0523[M-H-Glc-H_2_O]^-^,149.0617[M-H-Glc-CO_2_-H_2_O]^-^, 141.0189,123.0464,119.0356,105.0344,101.0250 | Iridoid |
| 30 | Ethyl caffeate | 8.95 | C_11_H_12_O_4_ | [M+HCOO]^-^ | 2.1 | 161.0329[M-H-C_2_H_5_O]^-^,135.0468,133.0282[M-H-C_2_H_5_O-CO]^-^ | Phenolic acid |
| 31 | Secologanin* | 9.03 | C_17_H_24_O_10_ | [M-H]^-^ | -3.6 | 341.1093[M-H-H_2_O-CO]^-^,179.0544[M-H-Glc-H_2_O-CO]^-^, 161.0444,149.0440,131.0336,119.0354,101.0247 | Iridoid |
| 32 | 7-epi-loganin | 9.2 | C_17_H_26_O_10_ | [M+HCOO]^-^ | 3.4 | 227.0932[M-H-Glc]^-^,209.0987[M-H-Glc-H_2_O]^-^,191.0563[M-H-Glc-2H_2_O]^-^,153.0697,149.0785,129.0559 | Iridoid |
| 33 | 7-O-ethyl sweroside | 9.23 | C_18_H_26_O_10_ | [M-H]^-^ | 2.8 | 175.0412[M-H-Glc-CH_3_CH_2_OH-H_2_O]^-^ | Iridoid |
| 34 | 8-epi-loganin | 9.23 | C_17_H_2_6O_10_ | [M+HCOO]^-^ | 4.6 | 227.0911[M-H-Glc]^-^,209.0809[M-H-Glc-H_2_O]^-^,191.0562[M-H-Glc-2H_2_O]^-^,133.0303,101.0250 | Iridoid |
| 35 | 5-(p-Coumaryl) quinic acid | 9.32 | C_16_H_18_O_8_ | [M-H]^-^ | 3.2 | 191.0575[QA-H]^-^,173.0442[QA-H-H_2_O]^-^,163.0391[M-H-QA]^-^,127.0387[QA-H-2H_2_O-CO]^-^, 117.0323[PA-H_2_O-CO]^-^ | Phenolic acid |
| 36 | 3-O-caffeoylquinic acid methyl ester | 10.78 | C_17_H_20_O_9_ | [M-H]^-^ | 4.7 | 191.0571[QA-H]^-^,173.0469[M-H-CA-CH_3_OH]^-^,127.0401[M-H-CA-CH_3_OH-H_2_O-CO]^-^ | Phenolic acid |
| 37 | 3-O-ferulicoylquinic acid | 10.78 | C_17_H_20_O_9_ | [M-H]^-^ | 4.7 | 191.0571[QA-H]^-^,173.0469[QA-H-H_2_O]^-^,127.0401[QA-H-2H_2_O-CO]^-^,117.0360[M-H-QA-OCH_3_-CO]^-^ | Phenolic acid |
| 38 | Ferulic acid* | 11.42 | C_10_H_10_O_4_ | [M-H]^-^ | 0.15 | 149.0608[M-H-COO]^-^,133.0295[M-H-COOH-CH_3_]^-^ | Phenolic acid |
| 39 | Loganin* | 11.47 | C_17_H_26_O_10_ | [M+HCOO]^-^ | 6.1 | 389.1781[M-H]^-^,227.1144[M-H-Glc]^-^ | Iridoid |
| 40 | Vogeloside | 11.8 | C_17_H_24_O_10_ | [M+HCOO]^-^ | 4.7 | 175.0402[M-H-Glc-CH_3_OH-H_2_O]^-^,151.0358[M-H-Glc-C_4_H_6_O]^-^,149.0591[M-H-Glc-CH_3_OH-H_2_O-C_2_H_2_]^-^, 119.0347,101.0251 | Iridoid |
| 41 | Kingiside | 11.88 | C_17_H_24_O_11_ | [M-H]^-^ | 3.9 | 165.0569,149.0262[M-H-Glc-CH_3_-COO-H_2_O-CH_3_]^-^, 121.0311,119.0382,101.0257 | Iridoid |
| 42 | Secoxyloganin* | 11.9 | C_17_H_24_O_11_ | [M-H]^-^ | 3.9 | 149.0252[M-H-Glc-H_2_O-CH_3_OH-C_2_H_2_O]^-^,121.0308[M-H-Glc-H_2_O-CH_3_OH-C_2_H_2_O-CO]^-^ | Iridoid |
| 43 | Hyperoside* | 15.18 | C_21_H_20_O_12_ | [M-H]^-^ | 3.4 | 301.0387[M-H-Glc]^-^,283.0131[M-H-Glc-H_2_O]^-^, 151.0054,107.0174 | Flavonoid |
| 44 | Rutin* | 16.01 | C_27_H_30_O_16_ | [M-H]^-^ | -1.8 | 301.0374[M-H-Rha-Glc]^-^ | Flavonoid |
| 45 | Quercetin-7-*O*-glucoside | 16.56 | C_21_H_20_O_12_ | [M-H]^-^ | 5.8 | 301.0374[M-H-Glc]^-^,271.0265[M-H-Glc-CH_2_O]^-^,151.0033 | Flavonoid |
| 46 | Isoquercitrin* | 16.68 | C_21_H_20_O_12_ | [M-H]^-^ | 3.9 | 301.0379[M-H-Glc]^-^,151.0044 | Flavonoid |
| 47 | Luteolin-5-*O*-β-D- glucopyranoside | 17.08 | C_21_H_20_O_11_ | [M-H]^-^ | 2.2 | 285.0418[M-H-Glc]^-^ | Flavonoid |
| 48 | Luteoloside* | 17.17 | C_21_H_20_O_11_ | [M-H]^-^ | 3.3 | 285.0419[M-H-Glc]^-^ | Flavonoid |
| 49 | Lonicerin* | 17.98 | C_27_H_30_O_15_ | [M-H]^-^ | 0.01 | 285.0405[M-H-Glc-Rha]^-^ | Flavonoid |
| 50 | Hesperidin* | 18.04 | C_28_H_34_O_15_ | [M-H]^-^ | -2.8 | 301.036[M-H-Rha-Glc]^-^, 271.0265,255.0304,179.0090,151.0033 | Flavonoid |
| 51 | 1,3-*O*-dicaffeoylquinic acid* | 18.54 | C_25_H_24_O_12_ | [M-H]^-^ | 0.6 | 353.0821[M-H-CA]^-^,335.0821[M-H-CA-H_2_O]^-^,191.0567[M-H-2CA]^-^,179.0377[CA-H]^-^,173.0481[M-H-2CA-H_2_O]^-^,161.0268[CA-H-H_2_O]^-^,135.0613[CA-H-CO_2_]^-^ | Phenolic acid |
| 52 | Kaempferol-3-*O*-rutinoside* | 19.07 | C_27_H_30_O_15_ | [M-H]^-^ | 4.4 | 593.1570,285.0419[M-H-Rha-Glc]^-^ | Flavonoid |
| 53 | Caffeic acid methyl ester | 19.49 | C_10_H_10_O_4_ | [M-H]^-^ | 4.4 | 133.0305[M-H-CH_3_-COOH]^-^ | Phenolic acid |
| 54 | Isochlorogenic acid B* | 20.06 | C_25_H_24_O_12_ | [M-H]^-^ | -2.8 | 353.0924[M-H-CA]^-^,335.0793[M-H-CA-H_2_O]^-^,191.0559[M-H-2CA]^-^,179.0355,173.0458[M-H-2CA-H_2_O]^-^,161.0241[CA-H-H_2_O]^-^,155.0346[M-H-2CA-2H_2_O]^-^, 135.0459[CA-H-CO]^-^ | Phenolic acid |
| 55 | 1,5-*O*-dicaffeoylquinic acid | 20.24 | C_25_H_2_4O_12_ | [M-H]^-^ | 5.6 | 353.0910[M-H-CA]^-^,335.0816[M-H-CA-H_2_O]^-^,191.0575[M-H-2CA]^-^,179.0369[CA-H]^-^,173.0472[M-H-2CA-H_2_O]^-^, 161.0257[CA-H-H_2_O]-,135.0464[CA-H-CO_2_]^-^ | Phenolic acid |
| 56 | Astragalin* | 20.6 | C_21_H_20_O_11_ | [M-H]^-^ | 2.2 | 285.0428[M-H-Glc]^-^ | Flavonoid |
| 57 | Isochlorogenic acid A* | 20.64 | C_25_H_24_O_12_ | [M-H]^-^ | -3.1 | 353.0901[M-H-CA]^-^,191.0569[M-H-2CA]^-^,179.0353[CA-H]^-^, 173.0463[M-H-2CA-H_2_O]^-^,135.0457[CA-H-CO]^-^ | Phenolic acid |
| 58 | 1,4-*O*-dicaffeoylquinic acid | 20.67 | C_25_H_24_O_12_ | [M-H]^-^ | 6.2 | 353.0905[M-H-CA]^-^,191.0576[M-H-2CA]^-^,179.0366[CA-H]^-^, 173.0460[M-H-2CA-H_2_O]^-^,161.0247[CA-H-H_2_O]^-^, 135.0460[CA-H-CO]^-^ | Phenolic acid |
| 59 | Apigenin-7-*O*-rutinoside | 22.61 | C_27_H_30_O_14_ | [M-H]^-^ | -1.3 | 269.0490[M-H-Rha-Glc]^-^,191.0582 | Flavonoid |
| 60 | Rhoifolin* | 22.68 | C_27_H_30_O_14_ | [M-H]^-^ | 4.5 | 413.0799[M-H-Rha-H_2_O]^-^,269.0462[M-H-Rha-Glc]^-^ | Flavonoid |
| 61 | Isochlorogenic acid C* | 23.09 | C_25_H_24_O_12_ | [M-H]^-^ | -3.1 | 353.0904[M-H-CA]^-^,191.0575[M-H-2CA]^-^,179.0360[CA-H]^-^, 173.0463[M-H-2CA-H_2_O]^-^,155.0364[M-H-2CA-2H_2_O]^-^, 135.0464[CA-H-CO]^-^ | Phenolic acid |
| 62 | Centauroside | 24.52 | C_34_H_46_O_19_ | [M-H]^-^ | 0.3 | 725.2528[M-H-OCH_3_]^-^,595.1979[M-H-Glc]^-^,525.1657[M-H-Glc-CH_2_-C_2_O_2_]^-^,179.0565 | Iridoid |
| 63 | Coumaroyl caffeoylquinic acid | 24.81 | C_25_H_24_O_11_ | [M-H]^-^ | 4.8 | 353.0935[M-H-PA]^-^,191.0560[QA-H]^-^,179.0347[CA-H]^-^, 173.0431[QA-H-H_2_O]^-^,163.0410[PA-H]^-^,161.0258[CA-H-H_2_O]^-^,135.0444[CA-H-H_2_O-CO]^-^,119.0476 | Phenolic acid |
| 64 | Coumaroyl caffeoylquinic acid isomer | 24.82 | C_25_H_24_O_11_ | [M-H]^-^ | 5.4 | 353.0879[M-H-PA]^-^,319.0844[M-H-CA]^-^,191.0562[QA-H]^-^, 179.0353[CA-H]^-^,173.0427[QA-H-H_2_O]^-^,163.0407[PA-H]^-^, 161.0242[CA-H-H_2_O]^-^,135.0444[CA-H-H_2_O-CO]^-^, 127.0399[PA-H-2H_2_O]^-^,119.0476 | Phenolic acid |
| 65 | Flavoyadorinin-B | 27.56 | C_23_H_24_O11 | [M-H]^-^ | 5.2 | 313.0715[M-H-Glc]^-^,283.0531,279.0164,269.0429,255.030 | Flavonoid |
| 66 | Feruloyl caffeoylquinic acid | 28.03 | C_26_H_26_O_12_ | [M-H]^-^ | 5.3 | 353.0898[M-H-C_10_H_8_O_3_]^-^,191.0568[M-H-C_10_H_8_O_3_-CA]^-^, 179.0366[CA-H]^-^,173.0460[M-H-C_10_H_8_O_3_-CA-H_2_O]^-^, 161.0247[CA-H-H_2_O]^-^,155.0379[M-H-C_10_H_8_O_3_-CA-2H_2_O]^-^, 135.0460[CA-H-CO]^-^ | Phenolic acid |
| 67 | Quercetin* | 28.42 | C_15_H_10_O_7_ | [M-H]^-^ | 0.2 | 301.0367[M-H]^-^,193[M-H-ringB]^-^,151,121,107 | Flavonoid |
| 68 | 4,5-*O*-dicaffeoylquinic acid methyl ester* | 29.4 | C_26_H_26_O_12_ | [M-H]^-^ | 5.0 | 367.1076[M-H-CA]^-^,349.0978[M-H-CA-H_2_O]^-^, 179.03340[CA-H]^-^,161.0243[CA-H-H_2_O]^-^,135.0447[CA-H-CO_2_]^-^ | Phenolic acid |
| 69 | Luteolin* | 29.66 | C_15_H_10_O_6_ | [M-H]^-^ | 4.5 | 199.0399[M-H-H_2_O-C_4_H_4_O]^-^, 175.0405,151.0026,133.0300,121.0299,107.0151 | Flavonoid |
| 70 | Kaempferol* | 29.71 | C_15_H_10_O_6_ | [M-H]^-^ | -1.7 | 285.0401,215.0382[M-H-H_2_O-C_4_H_4_]^-^, 175.0425,151.0027,133.0295 | Flavonoid |
| 71 | 3,4,5-tricaffeoylquinic acid | 30.75 | C_34_H_30_O_15_ | [M-H]^-^ | 6.3 | 515.1256[M-H-CA]^-^,353.0907[M-H-2CA]^-^,335.0794[M-H-2CA-H_2_O]^-^,191.0578[QA-H]^-^,179.0366[CA-H]^-^,173.0470[QA-H-H_2_O]^-^,161.0261[CA-H-H_2_O]^-^,135.0454[CA-H-H_2_O-C_2_H_2_]^-^ | Phenolic acid |
| 72 | Apigenin* | 30.9 | C_15_H_10_O_5_ | [M-H]^-^ | 3.4 | 117.0357 | Flavonoid |
| 73 | Genistein | 31.06 | C_15_H_10_O_5_ | [M-H]^-^ | 3.4 | 201.0572[M-H-C_3_O_2_]^-^,159.0517[M-H-C_3_O_2_-C_2_H_2_O]^-^,151.0089,107.0107 | Flavonoid |
| 74 | Tricin | 31.09 | C_17_H_14_O_7_ | [M-H]^-^ | -0.1 | 329.6663[M-H]^-^,211 | Flavonoid |
| 75 | Diosmetin* | 31.2 | C_16_H_12_O_6_ | [M-H]^-^ | -2.6 | 283.0425,255.0315[M-H-C_2_H_40_]^-^, 227.0347,199.0434,151.0044,147.0041,133.0309,107.014 | Flavonoid |
| 76 | Chrysoeriol | 31.2 | C_16_H_12_O_6_ | [M-H]^-^ | 6.3 | 283.0425,255.0315[M-H-C_2_H_40_]^-^, 227.0347,199.0434,151.0044,147.0041,133.0309,107.014 | Flavonoid |
| 77 | Sweroside* | 31.22 | C_16_H_22_O_9_ | [M+HCOO]^-^ | 4.9 | 151.0809,149.0254[M-H-Glc-H_2_O-CO]^-^,125.0263[M-H-Glc-C_4_H_6_O]^-^,119.0062 | Iridoid |
| 78 | Prunetin | 33.06 | C_16_H_12_O_5_ | [M-H]^-^ | 5 | 268.0367[M-H-CH_3_]^-^,239.0354[M-H-CH_3_-CHO]^-^, 171.0526[M-H-CH_3_-CHO-C_3_O_2_]^-^ | Flavonoid |
| 79 | Cupressuflavone/Ochanaflavone | 33.29 | C_30_H_18_O_10_ | [M-H]^-^ | 6.4 | 519.0860[M-H-H_2_O]^-^,493.0825[M-H-H_2_O-C_2_H_2_]^-^, 469.0991[M-H-C_4_H_4_O]^-^,427.0991[M-H-C_6_H_6_O_2_]^-^, 269.0400[M-H-C_15_H_9_O_5_]^-^,130.9961 | Flavonoid |

Note: *: Reference substance; QA: quinic acid; CA: caffeic acid; PA: p-coumaric acid
