# Supplementary material for: Metabolomics characterizes the metabolic changes of Lonicerae Japonicae Flos under different salt stresses
Source: PLoS One. 2020 Dec 1;15(12):e0243111. doi: 10.1371/journal.pone.0243111 (PMC7707481; doi:10.1371/journal.pone.0243111)
Supplement: S2 Table — (DOCX) [file pone.0243111.s006.docx]

**S2 Table** The relative contents of differentially metabolites in LJF under different salt

| **Metabolites** | **0 mM** | | | | | **100 mM** | | | | | **200 mM** | | | | | **300 mM** | | | | |
| --- | --- | --- | --- | --- | --- | --- | --- | --- | --- | --- | --- | --- | --- | --- | --- | --- | --- | --- | --- | --- |
| Caffeic acid | 26242 | 26220 | 26945 | 27198 | 28047 | 20974 | 21071 | 21493 | 20990 | 21695 | 13241 | 13214 | 13438 | 13162 | 13546 | 22515 | 23049 | 22703 | 22792 | 23048 |
| Quinic acid | 10487 | 10586 | 12277 | 12670 | 12741 | 10232 | 10143 | 10277 | 10171 | 10219 | 6076 | 6064 | 6023 | 6054 | 6332 | 9585 | 9670 | 9735 | 9397 | 9385 |
| 1,3-O-dicaffeoylquinic acid | 41409 | 17465 | 15610 | 30746 | 31468 | 36981 | 14824 | 0 | 37630 | 0 | 15612 | 33826 | 37810 | 35349 | 35858 | 37737 | 37279 | 38009 | 36735 | 38576 |
| Cryptochlorogenic acid | 711 | 906 | 3459 | 3839 | 3741 | 628 | 413 | 510 | 543 | 590 | 385 | 556 | 346 | 494 | 558 | 420 | 550 | 527 | 408 | 462 |
| 3-O-caffeoylquinic acid methyl ester | 1572 | 1704 | 1849 | 1688 | 1869 | 2455 | 2430 | 2332 | 2371 | 2554 | 1525 | 1428 | 827 | 1623 | 698 | 2983 | 3172 | 3168 | 3013 | 3299 |
| Feruloyl caffeoylquinic acid | 6102 | 5975 | 4460 | 4307 | 3794 | 8571 | 8175 | 8968 | 8572 | 9008 | 11666 | 11157 | 12243 | 10533 | 10599 | 12970 | 12187 | 12986 | 12574 | 12248 |
| 5-(p-Coumaryl) quinic acid | 12422 | 11872 | 13782 | 14124 | 12938 | 13570 | 13877 | 13799 | 12809 | 13485 | 12754 | 12856 | 12901 | 12782 | 12179 | 18499 | 18387 | 18998 | 19015 | 19368 |
| Coumaroyl caffeoylquinic acid | 59100 | 57178 | 45020 | 39894 | 44524 | 53650 | 49510 | 51508 | 24644 | 49649 | 50998 | 51278 | 53283 | 48814 | 52540 | 50242 | 51067 | 53024 | 53649 | 57738 |
| Isochlorogenic acid A | 9503 | 1104 | 8182 | 4450 | 7497 | 930 | 5684 | 1051 | 926 | 800 | 722 | 957 | 633 | 4458 | 504 | 4743 | 8410 | 451 | 0 | 4305 |
| Chlorogenic acid | 4889 | 4898 | 4829 | 4805 | 4865 | 5793 | 5641 | 5735 | 5522 | 5844 | 6127 | 6260 | 6568 | 6353 | 6296 | 4848 | 4878 | 4878 | 5025 | 4871 |
| 3-O-ferulicoylquinic acid | 0 | 0 | 0 | 0 | 0 | 0 | 0 | 0 | 38964 | 0 | 0 | 0 | 0 | 0 | 0 | 0 | 0 | 0 | 0 | 0 |
| Ferulic acid | 2432 | 2510 | 2539 | 2571 | 2472 | 2139 | 2126 | 2179 | 2198 | 2196 | 1591 | 1618 | 1613 | 1622 | 1600 | 2339 | 2320 | 2327 | 2332 | 2258 |
| Isochlorogenic acid C | 12056 | 11017 | 8108 | 7541 | 7837 | 1872 | 10157 | 10324 | 9609 | 10472 | 2000 | 9116 | 9536 | 1094 | 9141 | 1464 | 1544 | 0 | 1765 | 18078 |
| Neochlorogenic acid | 42018 | 41619 | 41516 | 39009 | 39163 | 40428 | 40845 | 40439 | 40218 | 40530 | 35927 | 35628 | 36485 | 35712 | 36797 | 37847 | 37374 | 37816 | 39953 | 37411 |
| Caffeic acid methyl ester | 0 | 8177 | 28 | 3088 | 0 | 7851 | 2891 | 7806 | 7436 | 7783 | 6945 | 6725 | 7478 | 3483 | 7110 | 3494 | 0 | 7463 | 7539 | 3575 |
| 1,4-O-dicaffeoylquinic acid | 391 | 373 | 450 | 1772 | 1770 | 2028 | 1745 | 1776 | 3004 | 1979 | 179 | 411 | 1654 | 1653 | 1633 | 332 | 3033 | 1778 | 2909 | 1833 |
| Ethyl caffeate | 2930 | 2776 | 4918 | 5222 | 4801 | 2122 | 2205 | 2056 | 2077 | 2344 | 749 | 765 | 801 | 752 | 777 | 2470 | 2359 | 2747 | 2635 | 2465 |
| Proline | 396 | 382 | 458 | 436 | 452 | 316 | 306 | 302 | 356 | 0 | 0 | 145 | 153 | 0 | 0 | 0 | 218 | 219 | 0 | 209 |
| Tryptophan | 1006 | 1055 | 549 | 490 | 500 | 489 | 506 | 524 | 501 | 547 | 454 | 475 | 435 | 375 | 426 | 726 | 689 | 673 | 733 | 706 |
| Adenine | 2019 | 2070 | 2852 | 3124 | 3330 | 1988 | 1945 | 2077 | 1847 | 1971 | 720 | 669 | 680 | 639 | 519 | 1941 | 1878 | 1849 | 1905 | 1813 |
| Uridine | 3062 | 3096 | 3268 | 3598 | 3569 | 3294 | 3033 | 3153 | 3098 | 3092 | 4222 | 4280 | 4366 | 4584 | 4220 | 5130 | 4763 | 4689 | 5081 | 4992 |
| Tyrosine | 1184 | 1237 | 971 | 969 | 974 | 1977 | 1932 | 1994 | 1952 | 1941 | 2361 | 2376 | 2331 | 2508 | 2341 | 1897 | 1740 | 1761 | 1782 | 1775 |
| Kingiside | 573 | 450 | 625 | 478 | 613 | 4046 | 189 | 4062 | 3857 | 263 | 212 | 163 | 145 | 208 | 374 | 371 | 389 | 371 | 331 | 319 |
| Luteoloside | 2283 | 2080 | 2159 | 2199 | 2013 | 1754 | 3457 | 1657 | 3256 | 3664 | 2763 | 2816 | 3124 | 2918 | 2909 | 3413 | 3639 | 3459 | 3488 | 3526 |
| Isoquercitrin | 3675 | 3521 | 3391 | 3082 | 2791 | 2357 | 434 | 2589 | 263 | 373 | 302 | 241 | 272 | 324 | 276 | 230 | 375 | 308 | 252 | 322 |
| Genistein | 459 | 386 | 356 | 355 | 429 | 441 | 452 | 615 | 504 | 448 | 372 | 361 | 389 | 370 | 352 | 498 | 631 | 492 | 478 | 519 |
| Luteolin-5-O-glucopyranoside | 3675 | 3521 | 3391 | 3082 | 2791 | 2357 | 434 | 2589 | 263 | 373 | 302 | 241 | 272 | 324 | 276 | 230 | 375 | 308 | 252 | 322 |
| Tricin | 459 | 386 | 356 | 355 | 429 | 441 | 452 | 615 | 504 | 448 | 372 | 361 | 389 | 370 | 352 | 498 | 631 | 492 | 478 | 519 |
| Astragalin | 9503 | 1104 | 8182 | 4450 | 7497 | 930 | 5684 | 1051 | 926 | 800 | 722 | 957 | 633 | 4458 | 504 | 4743 | 8410 | 451 | 0 | 4305 |
| Quercetin | 1884 | 1843 | 1399 | 1325 | 1189 | 2673 | 2739 | 2812 | 2675 | 2710 | 3712 | 3519 | 0 | 0 | 3311 | 4181 | 3957 | 4243 | 4026 | 3787 |
| Lonicerin | 5411 | 5021 | 419 | 4611 | 5769 | 5795 | 6219 | 6202 | 754 | 796 | 0 | 541 | 300 | 5377 | 348 | 415 | 462 | 0 | 450 | 457 |
| Hesperidin | 5411 | 5021 | 419 | 4611 | 5769 | 5795 | 6219 | 6202 | 754 | 796 | 0 | 541 | 300 | 5377 | 348 | 415 | 462 | 0 | 450 | 457 |
| Hyperoside | 3793 | 8569 | 6849 | 6713 | 6856 | 8096 | 7708 | 8251 | 7791 | 8354 | 6776 | 7131 | 7357 | 3513 | 6642 | 9652 | 9759 | 9266 | 0 | 10497 |
| Quercetin-7-O-glucoside | 1167 | 471 | 401 | 820 | 815 | 845 | 1810 | 2028 | 1788 | 0 | 2330 | 1035 | 2539 | 2525 | 2535 | 2228 | 2235 | 2266 | 2383 | 2230 |
| Centauroside | 4496 | 5191 | 5595 | 4508 | 4649 | 5231 | 5304 | 5741 | 4853 | 6233 | 4147 | 5435 | 14033 | 5221 | 5500 | 8638 | 19850 | 7917 | 8689 | 20087 |
| Flavoyadorinin-B | 1308 | 1786 | 1870 | 2100 | 2350 | 1566 | 1542 | 1705 | 3408 | 0 | 0 | 0 | 3583 | 0 | 0 | 0 | 0 | 0 | 0 | 4414 |
| Lonijaposide B | 1573 | 1598 | 1114 | 1111 | 1125 | 1421 | 1263 | 1364 | 0 | 1384 | 1398 | 1346 | 1462 | 1294 | 3031 | 1415 | 1430 | 1506 | 0 | 1689 |
| 8-epi-loganin | 2467 | 2394 | 2369 | 2241 | 2294 | 2404 | 2284 | 2393 | 2380 | 2358 | 2553 | 2484 | 2620 | 2536 | 2552 | 2217 | 2166 | 2199 | 2141 | 2142 |
| Secoxyloganin | 12422 | 11872 | 13782 | 14124 | 12938 | 13570 | 13877 | 13799 | 12809 | 13485 | 12754 | 12856 | 12901 | 12782 | 12179 | 18499 | 18387 | 18998 | 19015 | 19368 |
| Secologanin | 2647 | 2454 | 2965 | 2916 | 2748 | 2981 | 2889 | 2807 | 2743 | 2986 | 2749 | 2733 | 2716 | 2713 | 2656 | 3941 | 3967 | 4182 | 4113 | 3979 |
| Loganin | 6323 | 6479 | 1985 | 1433 | 1665 | 9341 | 3975 | 9526 | 0 | 9501 | 6352 | 6718 | 6328 | 6243 | 6313 | 6420 | 0 | 5891 | 6463 | 2955 |
| 8-epi-loganin acid | 8016 | 8117 | 8508 | 8847 | 8599 | 7577 | 6996 | 7069 | 7419 | 7095 | 5565 | 5811 | 5974 | 5483 | 5629 | 7249 | 7912 | 7967 | 7596 | 8197 |
| Morroniside | 24210 | 24475 | 22235 | 20383 | 21296 | 20385 | 21447 | 21999 | 20955 | 22540 | 17786 | 18731 | 19256 | 18381 | 18830 | 18538 | 18322 | 18544 | 17517 | 18713 |
| Swertiamarine | 6550 | 6410 | 7067 | 6274 | 6120 | 10571 | 10339 | 10027 | 10339 | 10768 | 11867 | 11535 | 11802 | 11564 | 11839 | 11699 | 11838 | 12848 | 12926 | 12816 |
| Loganic acid | 13043 | 12725 | 12215 | 11179 | 10498 | 10864 | 11484 | 12842 | 10450 | 11377 | 7294 | 7473 | 7982 | 7307 | 7697 | 9546 | 9408 | 9448 | 10401 | 10004 |
| Vogeloside | 2823 | 2748 | 1125 | 673 | 658 | 5933 | 5828 | 6570 | 5814 | 6939 | 2084 | 1976 | 2215 | 2214 | 2106 | 2227 | 2682 | 2468 | 2220 | 2557 |
| Sweroside | 10 | 5 | 21 | 5 | 737 | 12 | 33 | 59 | 0 | 0 | 0 | 0 | 0 | 0 | 0 | 0 | 0 | 0 | 0 | 21 |
